# Sleep deprivation-induced anxiety-like behaviors are associated with alterations in the gut microbiota and metabolites

**DOI:** 10.1128/spectrum.01437-23

**Published:** 2024-02-29

**Authors:** Nana Zhang, Xuefeng Gao, Donghao Li, Lijuan Xu, Guanzhou Zhou, Mengqi Xu, Lihua Peng, Gang Sun, Fei Pan, Yan Li, Rongrong Ren, Ruolan Huang, Yunsheng Yang, Zikai Wang

**Affiliations:** 1 Medical School of Chinese PLA, Beijing, China; 2 Department of Gastroenterology and Hepatology, The First Centre of Chinese PLA General Hospital, Beijing, China; 3 Shenzhen Key Laboratory of Gastrointestinal Microbiota and Disease, Integrative Microecology Clinical Center, Shenzhen Hospital of Southern Medical University, Shenzhen, Guangdong, China; 4 Shenzhen Clinical Research Center for Digestive Disease, Shenzhen Hospital of Southern Medical University, Shenzhen, Guangdong, China; 5 The Clinical Innovation & Research Center, Shenzhen Hospital, Southern Medical University, Shenzhen, Guangdong, China; 6 Department of Neurology, Shenzhen University Clinical Research Center for Neurological Diseases, Shenzhen University General Hospital, Shenzhen, China; State Key Laboratory of Food Science and Resources, Nanchang, China

**Keywords:** sleep deprivation, gut microbiota, metabolomics, anxiety, probiotics

## Abstract

The present study aimed to characterize the gut microbiota and serum metabolome changes associated with sleep deprivation (SD) as well as to explore the potential benefits of multi-probiotic supplementation in alleviating SD-related mental health disorders. Rats were subjected to 7 days of SD, followed by 14 days of multi-probiotics or saline administration. Open-field tests were conducted at baseline, end of SD (day 7), and after 14 days of saline or multi-probiotic gavage (day 21). Metagenomic sequencing was conducted on fecal samples, and serum metabolites were measured by untargeted liquid chromatography tandem-mass spectrometry. At day 7, anxiety-like behaviors, including significant decreases in total movement distance (*P* = 0.0002) and staying time in the central zone (*P* = 0.021), were observed. In addition, increased levels of lipopolysaccharide (LPS; *P* = 0.028) and decreased levels of uridine (*P* = 0.018) and tryptophan (*P* = 0.01) were detected in rats after 7 days of SD. After SD, the richness of the gut bacterial community increased, and the levels of *Akkermansia muciniphila*, *Muribaculum intestinale*, and *Bacteroides caecimuris* decreased. The changes in the host metabolism and gut microbiota composition were strongly associated with the anxiety-like behaviors caused by SD. In addition, multi-probiotic supplementation for 14 days modestly improved the anxiety-like behaviors in SD rats but significantly reduced the serum level of LPS (*P* = 0.045). In conclusion, SD induces changes in the gut microbiota and serum metabolites, which may contribute to the development of chronic inflammatory responses and affect the gut-brain axis, causing anxiety-like behaviors. Probiotic supplementation significantly reduces serum LPS, which may alleviate the influence of chronic inflammation.

**IMPORTANCE:**

The disturbance in the gut microbiome and serum metabolome induced by SD may be involved in anxiety-like behaviors. Probiotic supplementation decreases serum levels of LPS, but this reduction may be insufficient for alleviating SD-induced anxiety-like behaviors.

## INTRODUCTION

Sleep deprivation (SD) occurs when one does not get enough sleep owing to various factors, such as circadian rhythm disturbance and poor sleeping habits (1). In the United States, 35% of adults have been reported to sleep less than 7 h during a 24-h day (2). SD affects the metabolism of individuals and is linked to obesity, diabetes, cardiovascular diseases, and other health issues related to metabolic disorders (3–6). Identifying metabolic changes helps uncover the potential mechanisms of the adverse effects of SD. Individuals suffering from SD have insulin resistance and altered serum lipoproteins, such as decreased circulating high-density lipoprotein (7, 8). Analysis of the plasma metabolome profile of individuals who suffered from acute SD revealed that 27 metabolites were higher than their sleep status (9). Moreover, SD-induced changes in the gut microbiota also contribute to metabolic diseases (10–12).

SD has been demonstrated to increase the risk of mental health disorders, including depression and anxiety (13, 14), and the potential mechanisms include neuroinflammation caused by the activated innate immune responses in the brain (15–18). Lipopolysaccharide (LPS) is the primary surface membrane component of Gram-negative bacteria, and it participates in host-pathogen interactions with the innate immune system (19). Increased circulating LPS has been identified as a mediator of SD-related systemic inflammation (20, 21). Rats and mice administered LPS show elevated inflammatory cytokine levels that mediate neuroinflammation and anxiety-like behavior (16, 22).

Several lines of evidence have suggested that the gut microbiota bidirectionally communicates with the brain through nerve, metabolism, and immune pathways, namely, the gut-microbiota-brain (GMB) axis (23). A previous study has suggested that anxiety and depression are affected by gut microbiota dysbiosis mediated by the GMB axis (24, 25). Studies have demonstrated that individuals with bipolar disorder exhibit an altered gut microbiome characterized by reduced diversity and decreased abundance of butyrate-producing bacteria, especially *Faecalibacterium* (26, 27). The circadian rhythm of the host regulates the gut microbiota, especially the feeding/fasting rhythm (28). Gut microbiota dysbiosis and disruption of circadian rhythms have been suggested to play a role in systemic inflammation and psychiatry disorders caused by acute 72-h SD (29).

Preliminary evidence has demonstrated the antidepressant and anxiolytic effects of probiotics (30). Notably, *Lactobacillus* spp. (such as *Lactobacillus acidophilus* and *Lactobacillus rhamnosus*) have been shown to regulate the level of neurotransmitters (such as γ-aminobutyric acid) in the cortex, thereby ameliorating anxiety- and depression-like behaviors in mice (31, 32). Supplementation with *Lactobacillus plantarum* has been reported to moderate SD-induced immune system stress (33). *Bacillus subtilis* may exert antidepressant and anti-anxiety functions by increasing serotonin levels through tryptophan production (34). *Bifidobacterium* supplementation has been shown to attenuate insulin resistance in rhesus macaques induced by long-term SD (35), and *Bifidobacterium licheniformis* has been shown to prevent depression-like and anxiety-like behaviors in chronic unpredictable mild stress model rats, potentially through increasing the short-chain fatty acid levels in the colon to alter the levels of the neurotransmitters in the brain (36). *Enterococcus faecium* produce dopamine from dietary sources of L-dopa, thereby alleviating depression- and anxiety-like behaviors (37). Thus, the combination of these probiotics may improve SD-induced psychological symptoms.

This present study aimed to explore the effects of SD on changes in the gut microbiota and serum metabolites as well as to explore their involvement in anxiety-like behavior. The effect of multi-probiotic (including *Lactobacillus acidophilus*, *Bacillus licheniformis*, *Bacillus subtilis*, and *Enterococcus faecium*) supplementation was also assessed in alleviating SD-induced anxiety-like behavior.

## MATERIALS AND METHODS

### Sleep deprivation rat model

Thirty 6-week-old male Sprague-Dawley rats were purchased from Beijing Vital River Laboratory Animal Technology Co., Ltd. (Beijing, China). All rats were fed water and food *ad libitum*. The room humidity was 55%–65% with a 12-h light/dark cycle (light phase from 7:00 a.m. to 7:00 p.m.). The Ethics Committee on Animal Experimentation of the Chinese PLA General Hospital approved the study design.

**Fig 1:**
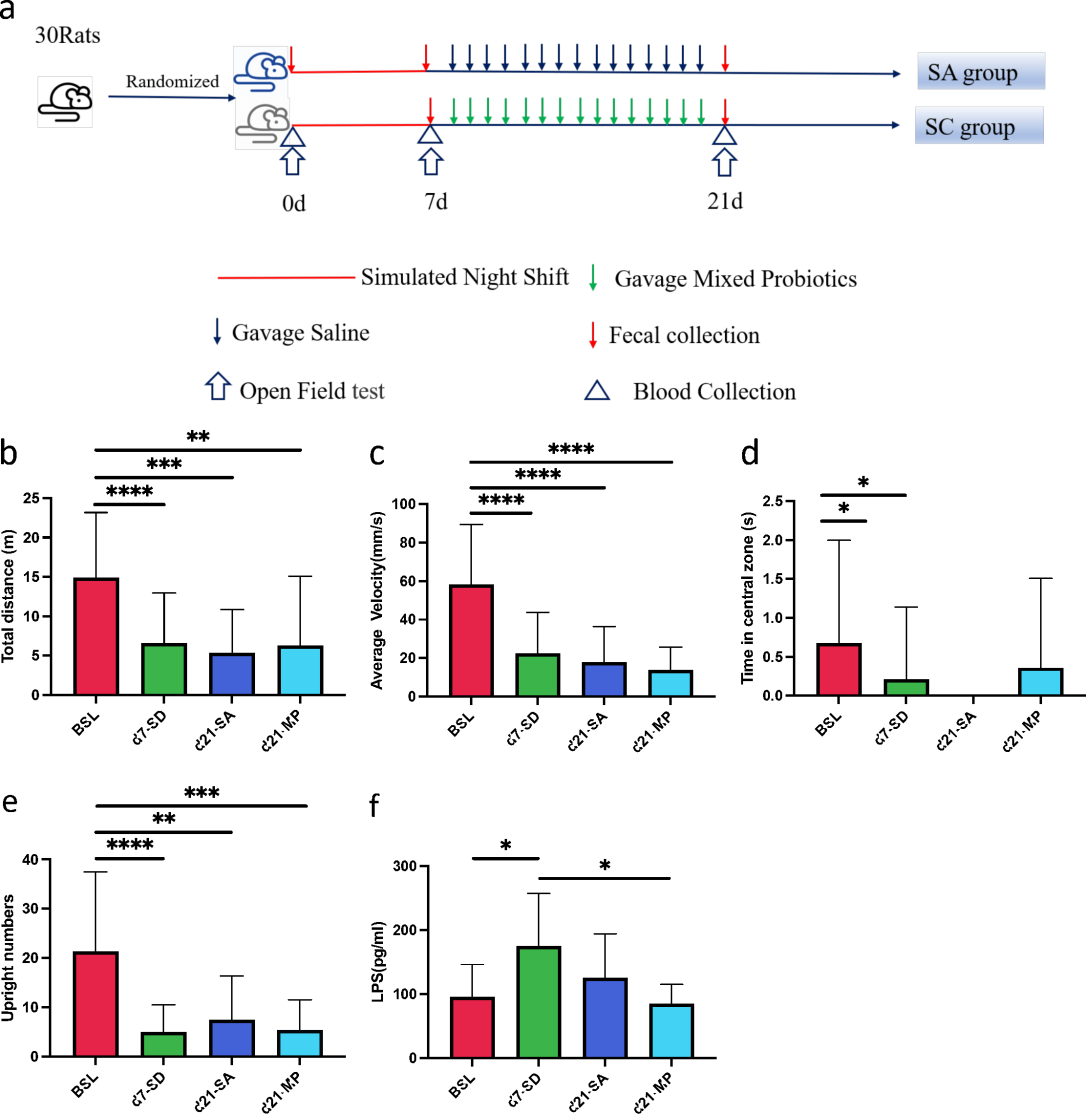
Rats show anxiety-like behaviors after sleep deprivation (SD) and respond to multi-probiotic supplementation. (a) Study protocols, (b) Total distance, (c) average velocity, (d) time in the central zone, (e) upright numbers, and (f) serum LPS levels of rats. Data are expressed as means ± SD. The significance of the comparison was determined using the Mann-Whitney *U* test. *P < 0.05, **P < 0.01, ***P < 0.001, and ****P < 0.0001. BSL, baseline (*N* = 30); 7d, SD day 7 (*N* = 29); SA-21d, saline supplementation for 14 days after SD (*N* = 15); SC-21d, multi-probiotic supplementation for 14 days after SD (*N* = 14).

After 1 week of adaption, 30 seven-week-old rats were randomly separated into the saline (SA) control group (SA group) and the multi-probiotic (MP) intervention group (MP group) (0 day). This study conducted SD by disturbing the normal circadian rhythm of rats (1). The sleeping habits of rats are opposite to those of humans, and forcing them to not sleep in the daytime disturbs their sleep-wake cycle, thus causing SD. All rats were forced to be active from 7:00 a.m. to 7:00 p.m. (12 h/day) for 7 days by using a sleep disturbance device (XR-XS 107; Shanghai Xinruan Information Technology Co., Ltd., China), which was a feeding cage equipped with a rod at the bottom and set for operating back and forth periodically (38). The operation interval was set at low frequency (30 s) to disturb the sleep of rats while avoiding increased physical activity/exercise. At the end of the SD period, the rats in the SA group were supplemented with saline, and the rats in the MP group were supplemented with multi-probiotics by gavage for 14 days (i.e., day 21). At 0, 7, and 21 days, open-field tests were conducted to evaluate the behavior performance, and serum and fecal samples were also collected. A flow chart of the experiment is shown in Fig. 1a. Knight et al. reported that cage mate fecal microbiomes become more homogeneous over time as rodents are coprophagic, suggesting that experiments should be replicated across multiple cages to control cage effects (39), and they also reported that single housing may stress mice and should be avoided. Therefore, the present study was conducted with a replicate across multiple cages with more than one rat per cage. After 7 days of SD, rats were transferred to individually ventilated cages (IVCs), and five rats were raised in one IVC. Regarding the coprophagy of rats, rats received SA or MP supplementation separately and were housed in different cages.

### Probiotic supplementation

After 7 days of SD, rats in the SA group were given normal saline (10 mL/kg, 4 mL per rat) by gavage from 2:30 to 3:00 p.m. Rats in the MP group were given mixed probiotics suspended in saline (a total volume of 10 mL/kg), including *Bifidobacterium* (≥1.0 × 10^6^/CFU)*, Lactobacillus acidophilus* (≥ 1.0 × 10^6^/CFU), *Enterococcus* (≥1.0 × 10^6^/CFU), *Bacillus licheniformis* (2.5 × 10^8^/CFU), *Bacillus subtilis* (5.0 × 10^7^/CFU), and *Enterococcus faecium* (4.5 × 10^8^/CFU). The *Bifidobacterium*, *Lactobacillus acidophilus*, and *Enterococcus* were from Bifid Triple Viable Capsules (Shanxi Jincheng Haisi Pharmaceutical Co., Ltd.; approval number: National Medicine Standard S19993065). The *Bacillus licheniformis* was from Zhengchangsheng (Shenyang No. 1 Pharmaceutical Co., Ltd.; approval number: National Medicine Standard S10950019). The *Bacillus subtilis* and *Enterococcus faecium* were from live combined *Bacillus subtilis* and *Enterococcus faecium* enteric-coated capsules (Beijing Hanmi Pharmaceutical Co., Ltd.; approval number: National Medicine Standard S20030087).

### Open-field tests

The open field was a 100 × 100 × 40 cm chamber, and the inner walls of the chamber were painted black. Before the tests, the rats were acclimated in the test room for at least 3 h. Rats were placed in the center of the bottom, and their spontaneous activities were recorded using a 1/3 SONY Super HAD CCD video for 5 min. The total distance moved, average velocities, time in the central zone, and the upright numbers were recorded using the Supermaze system (XR-XZ301, China, Shanghai Xinruan Information Technology Co., Ltd.). After finishing every test, the open field was cleaned with 75% alcohol to eliminate odors. At baseline (0 day), 7 days, and 21 days, the tests were conducted from 1:00 to 5:00 p.m. as previously reported by Andrade et al. (40), who reported that the behavior performance of rats is consistent during this time.

### Metagenomic sequencing and data processing

At baseline (0 day), 7 days, and 21 days, approximately 1 g of feces was collected per rat, placed into a 2-mL Eppendorf (EP) microcentrifuge tube, and stored at –80 ℃. The stool was collected by putting rats singly in a sterile can and waiting for defecation from 3:00 to 4:30 p.m. Fecal DNA was extracted using a QIAamp DNA Stool Mini Kit (Qiagen, USA) with the bead-beating step. The quality of extracted DNA was assessed using agarose gel electrophoresis. The genomic library was constructed using 1.5 µg of DNA fragmented using the Covaris E210 (Covaris, USA). According to the manufacturer’s instructions, metagenomic libraries were constructed using an Ovation Ultralow library systems V2 kit (NuGEN, San Carlos, CA). Next-generation sequencing (paired-end, 2 × 150 bp) was performed using the Illumina NovaSeq 6000 System.

Metagenomic sequencing data were processed using bioBakery 3 (41). The removal of low-quality reads and host (rat) sequences was performed using KneadData with default parameters. Taxonomic classification was generated using MetaPhlAn v3.0. The microbial species were annotated using clade-specific marker sequences.

### Blood collection and LPS measurement

The plasma samples were collected in ethylenediaminetetraacetic acid (EDTA) tubes from 3:00 to 4:30 p.m. without pre-fasting by cutting the tail and centrifuging at 3,000 rpm for 10 min. The upper serum was preserved in EP tubes and frozen at –80°C within 2 h for further metabolomic tests. Serum LPS was measured using a rat EDT ELISA Kit (The Blue Gene for Life Science Co., Ltd., China) following the manufacturer’s protocol. To avoid contamination of LPS from other sources, the EDTA tubes for whole blood collection were sterilized, and the EP tubes for collecting serum samples were also sterilized using an autoclave. The levels of LPS were measured according to the manufacturer’s protocol, and the labware was free of pyrogens and endotoxins.

### Untargeted liquid chromatography tandem-mass spectrometry-based metabolomic profiling

A total of 30 serum samples were included for metabolomic analysis. The metabolites were measured using ultrahigh performance liquid chromatography (1290 Infinity LC, Agilent Technologies) coupled to a quadrupole time-of-flight (AB Sciex TripleTOF 6600). The plasma samples were thawed at 4°C. To remove the serum protein, aliquots (100 µL) were mixed with 400 µL of cold methanol/acetonitrile (1:1, vol/vol) and then centrifuged for 15 min (14,000 × *g*, 4°C). The supernatant was dried in a vacuum centrifuge and redissolved in 100 µL of acetonitrile/water (1:1, vol/vol) solvent for further analysis. The metabolites were measured in electrospray ionization positive and negative modes using a 2.1 mm × 100 mm ACQUIY UPLC BEH 1.7 µm column (Waters, Ireland). The mobile phase A contained 25 mM ammonium acetate and 25 mM ammonium hydroxide in water, and mobile phase B contained acetonitrile. The following gradient was utilized: 85% B for 1 min, linearly reduced to 65% in 11 min, reduced to 40% in 0.1 min, maintained for 4 min, increased to 85% in 0.1 min, and a 5-min re-equilibration period. To monitor the stability and repeatability of instrument analysis, quality control (QC) samples were prepared by pooling 10 µL of each sample, and the pooled samples were analyzed together with the other samples. The QC samples were inserted regularly and analyzed after every five samples. The selection of samples during the operation was random, and the sample information was unknown to the operator.

Collection of Algorithms of MEtabolite pRofile Annotation was used to annotate isotopes and adducts. The accuracy m/z value compared compound identification of metabolites (<25 ppm) and MS/MS spectra with an in-house database established with available authentic standards. Features were filtered if their relative standard deviations were over 30% in QC samples. An interquartile range filter was applied to maintain the top maximum features. Sample normalization was performed using sums, and Pareto scaling was used to scale the metabolites.

### Statistical analysis

Behavior and serum index differences were analyzed using GraphPad Prism 9 (GraphPad Prism Software, Inc., San Diego, CA). As the samples were tied to time series comparisons, the principles reported by Festing et al. were utilized, and further multiple comparisons and Bonferroni correction were also conducted (42). The gut microbiota composition and diversity analysis were performed using MicrobimeAnalyst 5.0 (43). The Mann-Whitney *U* test was used to compare differences in gut microbiota alpha diversity among the groups. Linear discriminant analysis (LDA) effect size (LEfSe) was used to identify differential bacterial species between groups. One-way analysis of variance (ANOVA) was used to compare the relative abundances of taxa (transformed into a centered-log ratio) among the four groups. Statistical analysis of the metabolites was performed using MetaboAnalyst 5.0. Comparisons between the experimental groups were performed using Student *t*-test and one-way ANOVA, and *P* values were adjusted with the false discovery rate (FDR). Heatmap clustering analysis was performed using the Euclidian distance method with Ward’s clustering algorithm. Spearman’s rank correlation was used to analyze the associations among anxiety-like behaviors, gut bacterial taxa, and serum metabolites. Redundancy analysis (RDA) was applied to explore the association between movement indexes and the differential gut bacteria and serum metabolites induced by SD.

## RESULTS

### Sleep deprivation induces anxiety-like behaviors in rats, which are modestly improved by probiotic supplementation

After SD for 7 days, the open-field test was used to assess the behavior and general activity of rats (Fig. 1a). The total movement distance (*P* = 0.0002), average velocity (*P* < 0.0001), staying time in the central zone (*P* = 0.021), and upright numbers (*P* < 0.0001) were all significantly decreased after 7 days of SD compared to the baseline level (Fig. 1b through e). These findings suggested that SD induces anxiety-like behavior in rats.

After 14 days of saline (SA group) or multi-probiotic (MP group) gavage, the behaviors of the SD rats did not significantly recover in both groups. However, the mean total movement distance in the MP group was 6,256 ± 8,816 mm, which was slightly higher than the SA group (5,306 ± 5,539 mm; *P* = 0.73; Fig. 1b). The average velocity was 20.87 mm/s (SD ±29.39 mm/s) and 17.82 ± 18.62 mm/s in the MP and SA groups, respectively (Fig. 1c). The mean staying time in the central zone in the MP group was 0.36 ± 1.15 s and was 0 s for the SA group (Fig. 1d). The mean upright number in the MP group was 5.36 ± 6.17, which was slightly lower than that in the SA group (7.40 ± 8.90; Fig. 1e).

Because LPS has been suggested to induce anxiety- and depression-like behaviors in mice, serum LPS in rats was measured during the SD experiment (Fig. 1f). Serum LPS levels increased significantly from 95.01 ± 51.22 pg/mL at baseline to 174.70 ± 82.19 pg/mL after 7 days of SD (*P* = 0.028). After 14 days of MP supplementation, serum LPS levels decreased significantly in the MP group at 21 days (84.64 ± 30.25) compared to the levels in the SD group at 7 days (174.70 ± 82.19, *P* = 0.045). The serum LPS levels in the MP group were lower than those of the SA group (125.0 ± 68.97), although not significantly.

### The serum metabolites changed after SD and in response to probiotics supplementation

**Fig 2:**
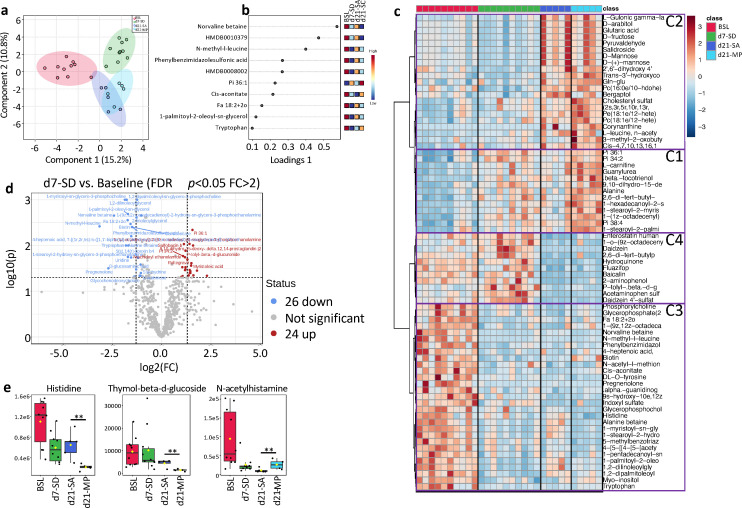
Changes in serum metabolites after sleep deprivation and mixed probiotics supplementation. (a) Sparse partial least squares discriminant analysis score plot and (b) loading plot of the serum metabolites. (c) The heatmap shows all serum metabolites in the experimental rats at baseline (BSL), 7 days post-SD (**d7**), and 14 days of gavage with saline (SA-21d) or mixed probiotics (MP-21d). Four patterns of metabolic changes were classified and indicated as clusters C1–C4. (d) Volcano plot showing the differential metabolites between 7 days post-SD and BSL (FDR <0.05 and fold change >2). Red represents the upregulated differential metabolites at 7 days, and blue represents the downregulated differential metabolites at 7 days. (e) Three metabolites with significantly differential serum levels between the 21d-SA and 21d-MP groups (*t*-test; *P* < 0.01 and fold change >2).

A total of 1,260 metabolites were detected in the rat serum by untargeted liquid chromatography coupled to tandem mass spectrometry (LC-MS/MS). Sparse partial least squares discriminant analysis revealed substantial changes in the serum metabolites after 7 days (d7) of SD (Fig. 2a through c). The separation between d7 of SD and baseline was clear (Fig. 2a), and 50 metabolites changed significantly in the serum of the SD group (FDR <0.05 and fold change >2), including 24 increased and 26 decreased metabolites, respectively (Fig. 2d; Table S1). Kyoto Encyclopedia of Genes and Genomes pathway enrichment analysis showed that SD significantly increased pyrimidine metabolism (*P* < 0.05; Fig. S1a).

Supplementation of either MP or SA for 14 days after SD introduced changes in the serum metabolites (Fig. 2a; Table S2). Different patterns of metabolic changes were observed during the 14 days of recovery. We classified the differentially expressed metabolites into four clusters (Fig. 2c): cluster 1, metabolites that moderately increased during SD and increased further after recovery; cluster 2, metabolites not affected by SD but increased during recovery; cluster 3, metabolites moderately decreased by SD and maintained at low levels during recovery; cluster 4, metabolites not affected by SD but decreased during recovery.

At 14 days after SD cessation, 10 metabolites significantly decreased in the SA group, and 1 metabolite significantly increased in the SA group (FDR <0.05 and fold change >2; Table S3). Compared to baseline, 19 higher and 38 lower serum metabolites were identified in the SA group (FDR <0.05 and fold change >2; Table S4). Rats in the SC group showed no restoration of metabolic homeostasis, with 23 metabolites (11 increased metabolites and 12 decreased metabolites) significantly different from baseline (FDR <0.05 and fold change >2; Table S5). MP did not significantly affect metabolites after SD (FDR >0.05). After 14 days of SA or MP supplementation, histidine and thymol-beta-d-glucoside were lower in the MP group than in the SA group (Fig. 2e), whereas N-acetylhistamine was higher in the MP group than in the SA group (Fig. 2e).

### Effects of SD and MP supplementation on changing the gut microbiota in rats

**Fig 3:**
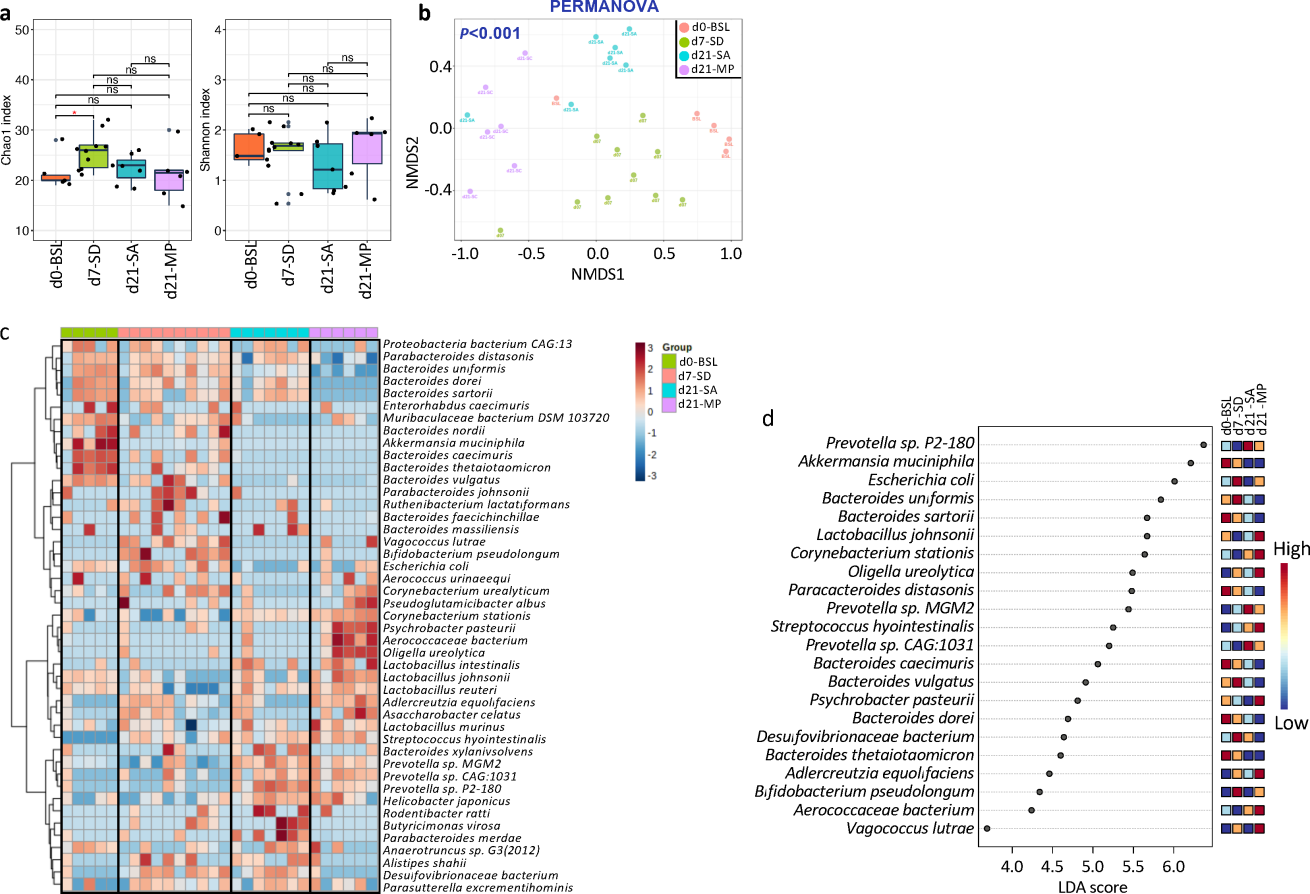
Changes in the gut microbiota after sleep deprivation and mixed probiotic supplementation. (a) At the species level, the alpha diversity of the gut microbiota was measured by the Chao1 index (richness) and Shannon index (diversity). The significance of the comparison was determined using the Mann-Whitney *U* test (*P < 0.05). (b) Beta diversity was visualized using non-metric multidimensional scaling with Bray-Curtis dissimilarity distances at the species level. Permutational multivariate analysis of variance was performed to assess the statistical significance among the four groups. (c) Heatmap showing the 74 different species identified using one-way ANOVA (FDR <0.005). (d) LEfSe analysis identified bacteria species that significantly differed (FDR <0.05 and LDA >3) among samples collected at baseline (BSL), 7 days post-SD (7d-SD), and 14 days of saline (21d-SA) or mixed probiotics (21d-MP) after SD.

Metagenomic analysis revealed that SD disturbed the gut microbiota homeostasis. The richness of the gut bacterial community (measured by the Chao1 index; *P* < 0.05) significantly followed SD, while the evenness (measured by the Shannon index) was not influenced (Fig. 3a). The gut bacterial richness dropped during the recovery period in both groups, and the decline was slightly sharper after MP supplementation, despite not significantly. Beta diversity analysis using non-metric multidimensional scaling revealed that the overall gut microbiota composition was influenced by the SD and MP supplementation permutational multivariate analysis of variance (PERMANOVA; *P* < 0.001; Fig. 3b). The gut microbiota was dominated by Verrucomicrobia (37%) at baseline, which disappeared after 7 days of SD with the enrichment of Proteobacteria (24%) (Fig. S2). Re-growth of Verrucomicrobia was not observed after 14 days of recovery.

Gut microbiota changes in response to SD and MP supplementation were also detected at the species level (Fig. 3c). LEfSe analysis showed that SD significantly increased the relative abundances of *Vagococcus lutrae*, *Adlercreutzia equolifaciens*, *Bifidobacterium pseudolongum*, *Desulfovibrionaceae bacterium*, and *Streptococcus hyointestinalis*; there were lower levels of *Akkermansia muciniphila*, *Muribaculum intestinale*, and *Bacteroides caecimuris* (FDR <0.05 and LDA >3; Fig. 3d). These changes were not reversed after 14 days of recovery (Fig. 3c). After the recovery period, differentially abundant bacterial species were measured between the MP and SA groups. *Romboutsia ilealis*, *Aerococcaceae bacterium*, *Adlercreutzia equolifaciens*, *Corynebacterium glutamicum, Psychrobacter pasteurii*, *Paenalcaligenes hominis*, *Oligella ureolytica*, *Corynebacterium stationis*, and *Lactobacillus johnsonii*. Lactobacillus *murinus* were more enriched in the MP group, while higher abundances of *Prevotella* sp. P2 180, *Parabacteroides distasonis*, *Prevotella* sp. MGM2, *Bacteroides sartorii*, *Parabacteroides merdae*, *Desulfovibrionaceae bacterium*, *Bacteroides dorei*, and *Bacteroides uniformis* were detected in SA group (Fig. 3c and d). During the recovery period, there was an increase in the relative abundance of *Prevotella* sp. MGM2, *Prevotella* sp. CAG 1031, *Prevotella* sp. P2 180, and *Helicobacter japonicus* in the SA and MP groups (Fig. 3c and d) suggesting that changes in these taxa were spontaneous or were caused by the saline solution or the gavage procedure.

### Anxiety behaviors are associated with changes in serum metabolism and gut microbiota

**Fig 4:**
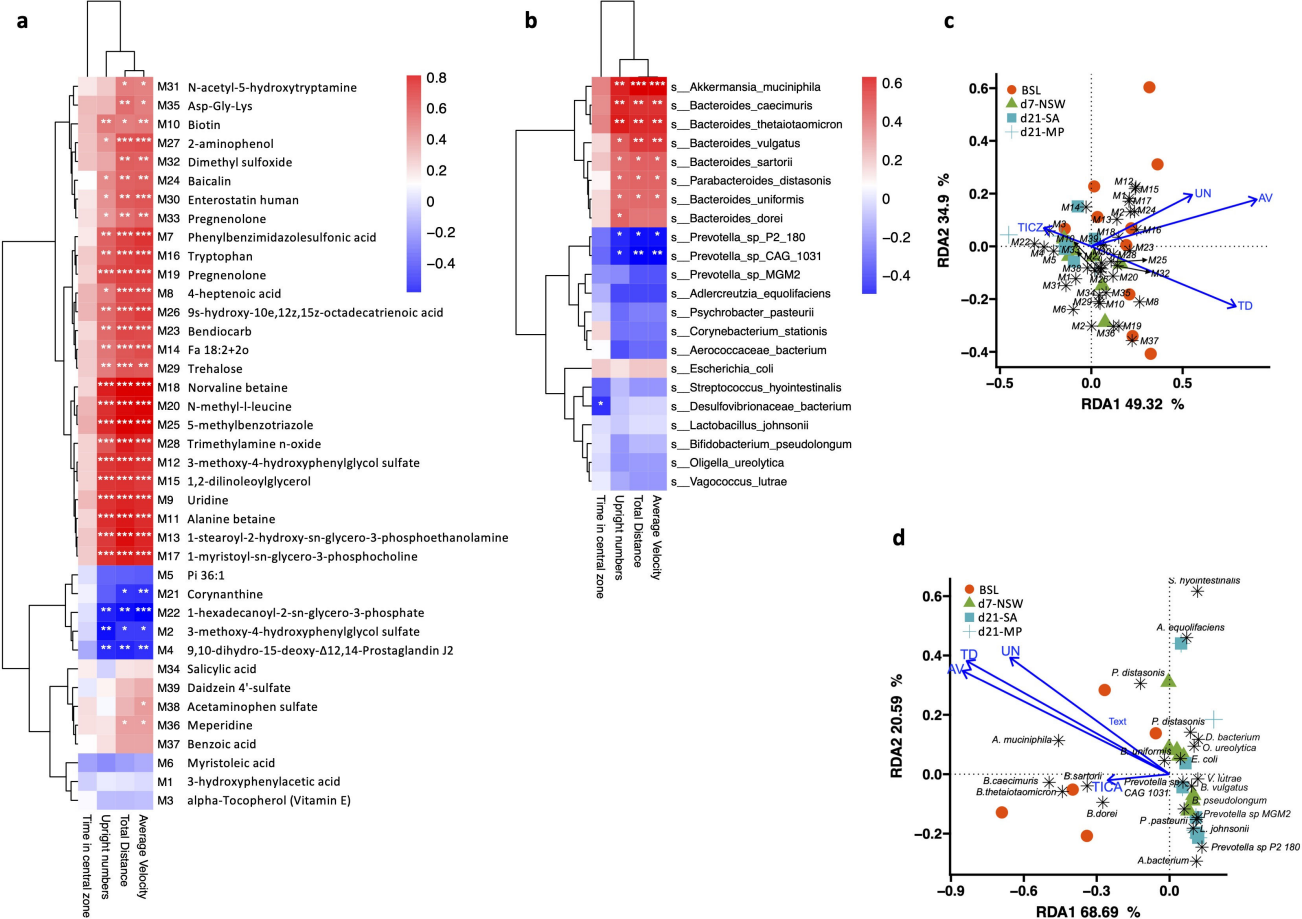
The severity of anxiety-like behaviors in rats after sleep deprivation is associated with the changes in serum metabolites and gut microbiota. Heatmaps showing that the (a) serum metabolites and (b) gut bacteria species significantly different from baseline had close relationships with the movement indexes. Significant Spearman correlations are indicated (*P < 0.05, **P < 0.01, and ***P < 0.001). RDA plots show the association of (c) the serum metabolites with four movement indexes and (d) the relationships between the differential taxa from baseline and the movement indexes. TD, total distances; AV, average velocity; UN, upright number; and TICZ, time in the central zone. The arrow length represents the strength of the correlation between the movement indexes and the serum metabolites or microbes.

We next explored the associations of behavior performance with changes in the serum metabolites and gut microbiota. We found that changes in the host metabolism and gut microbiota composition induced by SD might be potentially involved in developing anxiety-like behaviors. Spearman’s correlation analysis showed that the average velocity, total distance, and upright numbers were positively correlated with the serum levels of 22 metabolites (top 22 in the heatmap of Fig. 4a; Table S6; *P* < 0.05). Among these metabolites, phenylbenzimidazolesulfonic acid, uridine, alanine betaine, 1-palmitoyl-2-oleoyl-sn-glycerol, 1-stearoyl-2-hydroxy-sn-glycero-3-phosphoethanolamine, 1,2-dilinoleoylglycerol, tryptophan, 1-myristoyl-sn-glycero-3-phosphocholine, norvaline betaine, N-methyl-l-leucine, 5-methylbenzotriazole, and trimethylamine n-oxide showed positive correlations with these movement indexes (Spearman correlation coefficients *R* > 0.65). Corynanthine, 1-hexadecanoyl-2-sn-glycero-3-phosphate, 3-methoxy-4-hydroxyphenylglycol sulfate, 9,10-dihydro-15-deoxy-Δ12, and 14-prostaglandin J2 were inversely correlated with the average velocity, total distance, and upright numbers (Fig. 4a).

Correlations were also observed between the differential gut bacteria and the altered movement behaviors (Fig. 4b; Table S7). The average velocity, total distance, and upright numbers positively correlated with *Akkermansia muciniphila*, *Bacteroides caecimuris*, *Bacteroides thetaiotaomicron*, *Bacteroides vulgatus*, *Bacteroides sartorii*, *Parabacteroides istasonis*, and *Bacteroides uniformis* (Fig. 4b; Table S3; Spearman *P* < 0.05). Notably, *Akkermansia muciniphila* showed the strongest correlations with total distance (*R* = 0.63, *P* < 0.001), average velocity (*R* = 0.63, *P* < 0.001), and upright numbers (*R* = 0.57, *P* < 0.01). Two *Prevotella* spp. (*Prevotella* sp. P2-180 and *Prevotella* sp. CAG:1031) were negatively correlated with these movement indexes (Fig. 4b).

These relationships between movement, serum metabolites, and gut microbiota were also accessed using redundancy analysis. The total distance, average velocity, and upright numbers were strongly associated with the serum metabolites or gut microbiota, and the relation between time in the central zone and serum metabolites or gut microbiota was weak compared to the other associations (Fig. 4c and d).

## DISCUSSION

The present study demonstrated that SD induces anxiety-like behavior in rats, agreeing with human and other animal studies (44–48). The induced anxiety-like behaviors were sustained after 14 days of recovery, suggesting that the effects of SD on psychology may last for a long time even if the SD ended. Similarly, it has been reported that the degradation of performance patterns caused by SD persists for days even after restoring normal sleep (49). Another study has demonstrated that the mood of adolescents was impaired by a week of partial SD and was not fully recovered after two nights of normal sleep (50). Thus, some physical and phycological changes induced by SD may be irreversible and need further intervention to improve the recovery.

In the present study, LPS was increased significantly in the serum of rats after SD, which may be involved in the development of chronic inflammatory responses and contribute to the occurrence of anxiety-like behaviors. Previous studies have reported that peripheral low-grade inflammation is increasingly observed in patients with psychiatric disorders (51–54). For example, elevated concentration of C-reactive protein (CRP; >3 mg/L) has been detected in 21%–34% of patients with depression, along with increased concentrations of IL-6 and other inflammatory cytokines in blood and in cerebrospinal fluid (51–54). In the present study, SD increased the serum level of LPS, a biologically active substance in the outer membrane of Gram-negative bacteria, which are known to activate innate immunity and promote inflammatory responses (55). Artificially increasing peripheral LPS levels induces neuroinflammation (56), which causes anxiety-like behavior via the immune-kynurenine pathway or modulating neuronal plasticity (15, 16, 57). In the present study, the relative abundances of some Gram-negative bacteria (*Akkermansia muciniphila* and *Bacteroides caecimuris*) were decreased after SD, while those of some Gram-positive bacteria (*Streptococcus hyointestinalis*, *Adlercreutzia equolifaciens*, *Bifidobacterium pseudolongum*, *Aerococcaceae bacterium*, and *Vagococcus lutrae*) increased after SD. Because the circadian rhythm disruption related to SD causes gut barrier impairment (58–60), the translocation of Gram-negative bacteria from the gut to the circulation may explain their decreased levels in the intestine. An increase in Gram-negative bacteria and LPS production induced by SD may be involved in the development of anxiety-like behaviors. In the present study, there was a decreased relative abundance of *Akkermansia muciniphila* in SD rats. Notably, *Akkermansia muciniphila* has been suggested to play critical roles in multiple neuropsychiatric disorders through the GMB axis, including depression, anxiety, Alzheimer’s disease, cognitive impairment, substance use disorders, amyotrophic lateral sclerosis, multiple sclerosis, autism spectrum disorders, epilepsy, Parkinson’s disease, and stroke (61). Given the immunomodulatory and anti-inflammatory effects of *Akkermansia muciniphila* (62–64), we proposed that SD induces an increase in LPS-producing bacteria and a reduction in beneficial microbes, resulting in peripheral low-grade inflammation and intestinal barrier injury, thereby participating in the development of anxiety- and depression-like behaviors.

Growing evidence has indicated a link between host metabolism and circadian rhythms, suggesting that alterations in the metabolism may also be involved in SD-induced anxiety-like behaviors (65). In the present study, four clusters of metabolites that changed in different trajectories during SD and recovery stages were identified, and some of the varied metabolites may influence host psychological status and contribute to the development of anxiety-like behaviors. The present data revealed that upregulated pyrimidine metabolism in SD rats may be linked to anxiety-like behaviors, which agreed with recent studies, reporting that depression is related to purine and pyrimidine metabolism disorders (66). Among the decreased metabolites after SD, uridine has been identified as a neuroprotective factor that improves memory and suppresses depression (67, 68). As the availability of serotonin (5-hydroxytryptophan) depends on tryptophan, dietary intake of tryptophan is essential for maintaining mental health and well-being (69). In addition, tryptophan deficiency has been shown to cause depression, and tryptophan supplementation may alleviate anxiety and improve mood (70). Thus, a reduced level of tryptophan in SD rats may also contribute to anxiety-like behaviors. Moreover, the present data revealed that a decreased serum level of trimethylamine-N-oxide (TMAO) correlated with anxiety-like behaviors in SD rats. Some studies have reported that animals or humans with depression or anxiety disorders have higher TMAO levels than healthy controls, which may cause neuroinflammation (71, 72). However, decreasing TMAO levels has no significant influence on anxiety, depression-like behaviors, and memory formation in adult mice (73). Thus, further investigation is required to determine the role of TMAO in the development of psychiatric, cognitive, and behavioral disorders.

It has been reported that 14 days of probiotic supplementation moderates the impact of anticipatory stress on the immune system in the time prior to the night shift (74). However, improvement in depression or anxiety behaviors has not been reported. In the present study, 14 days of MP supplementation did not thoroughly recover the anxiety-like behaviors caused by SD. Clinical trials have suggested that probiotic administration lasting longer than a month yields small but significant effects for patients with depression, but the impact of probiotics does not differ from placebo (30). Thus, the potential therapeutic function of probiotics for depression and anxiety should be further evaluated by randomized clinical trials with psychiatric samples.

The present study had several limitations. Firstly, there was no control group without gavage; therefore, it was impossible to investigate the impact of saline on metabolism or gut microbiota. However, Zhang et al. reported that the gut microbiota of rats during adulthood is fairly stable without other interventions (75). Based on this evidence, we assumed that the gut microbiota of rats with normal sleeping cycles on the same day (7-day SD) was similar to baseline and that the before-and-after study was a feasible research design. Secondly, some physiological variables of rats, such as sleep duration, markers of circadian timing, and timing of food intake, were not measured in the present study. Thirdly, the serum and fecal samples were collected from different animals, complicating the detection of potential bacterial taxa involved in specific metabolic changes. Finally, the time required to recover from anxiety associated with SD without any intervention was not measured.

In conclusion, the present study provided evidence that SD induces anxiety disorders and that increased serum LPS may play an important role. The dysbiosis of gut microbiota and changes in serum metabolism show significant correlations with SD-induced anxiety behaviors, and the mechanism may involve a chronic inflammation response of the gut that affects the gut-brain axis. Moreover, probiotic supplementation has limited ability to improve SD-induced anxiety-like behaviors; however, it significantly reduces the production of LPS, which may reduce the influence of chronic inflammation.

## Data Availability

The raw data of metagenomic sequencing have been uploaded to the Sequence Read Archive database (https://trace.ncbi.nlm.nih.gov/Traces/home/) and are available for download via accession number PRJNA1049576.

## SUPPLEMENTAL MATERIAL

Fig. S1 spectrum.01437-23-s0001.tiff Changes in metabolic functional pathways caused by sleep deprivation.

Fig. S2 spectrum.01437-23-s0002.tiff Changes in the relative abundances of gut bacteria phyla after SD, and 14 days of saline or mixed probiotics gavage.

Supplemental legends spectrum.01437-23-s0003.docx Legends for Fig. S1 and S2 and Tables S1 to S7.

Table S1 spectrum.01437-23-s0004.xlsx Different metabolites between baseline day and 7-day sleep deprivation.

Table S2 spectrum.01437-23-s0005.xlsx Different metabolites among baseline day, 7-day sleep deprivation, and 14-day saline or mixed probiotic supplementation.

Table S3 spectrum.01437-23-s0006.xlsx Different metabolites between 7-day sleep deprivation (d7-SD) and 14-day saline supplementation (d21-SA).

Table S4 spectrum.01437-23-s0007.xlsx Different metabolites between baseline day and 14-day saline supplementation (d21-SA).

Table S5 spectrum.01437-23-s0008.xlsx Different metabolites between baseline day and 14-day mixed probiotic supplementation (d21-MP).

Table S6 spectrum.01437-23-s0009.xlsx Spearman's correlation analysis between movement behaviors and serum metabolites.

Table S7 spectrum.01437-23-s0010.xlsx Spearman's correlation analysis between movement behaviors and gut microbes.

ASM does not own the copyrights to Supplemental Material that may be linked to, or accessed through, an article. The authors have granted ASM a non-exclusive, world-wide license to publish the Supplemental Material files. Please contact the corresponding author directly for reuse.
