# Supplementary figures and images for: Sleep deprivation-induced anxiety-like behaviors are associated with alterations in the gut microbiota and metabolites
Source: Microbiol Spectr. 2024 Feb 29;12(4):e01437-23. doi: 10.1128/spectrum.01437-23 (PMC10986621; doi:10.1128/spectrum.01437-23)

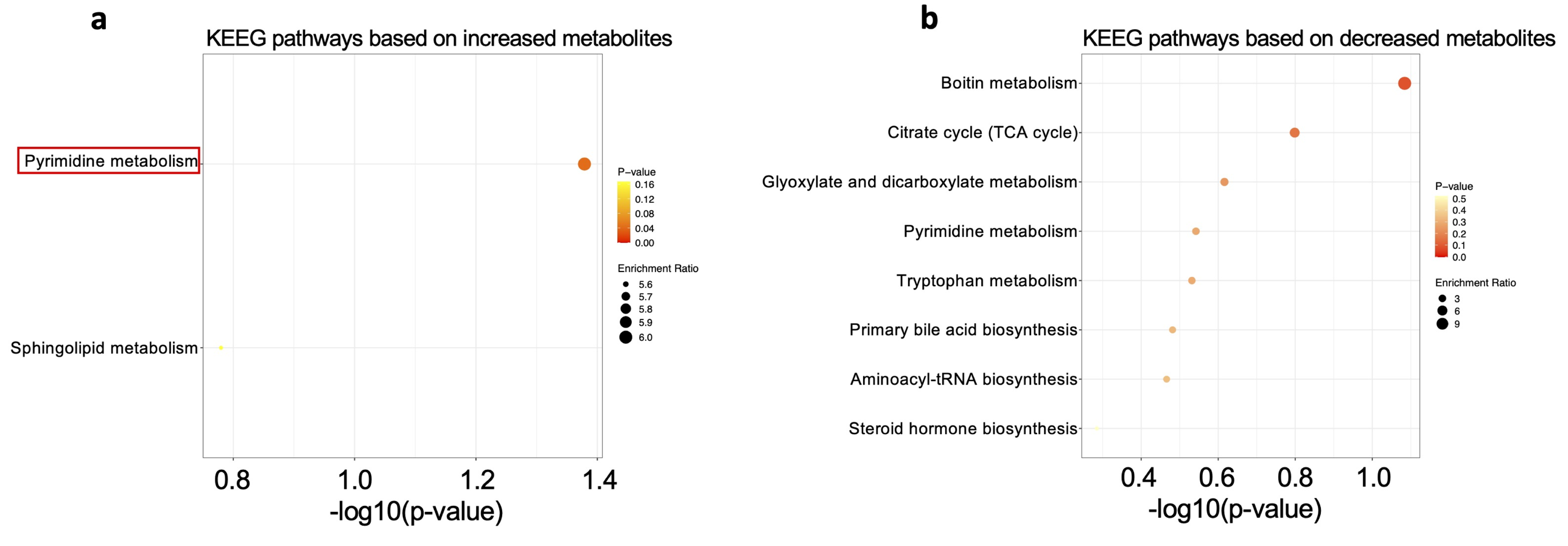

Supplement: Fig. S1 — Changes in metabolic functional pathways caused by sleep deprivation. [file spectrum.01437-23-s0001.tiff]

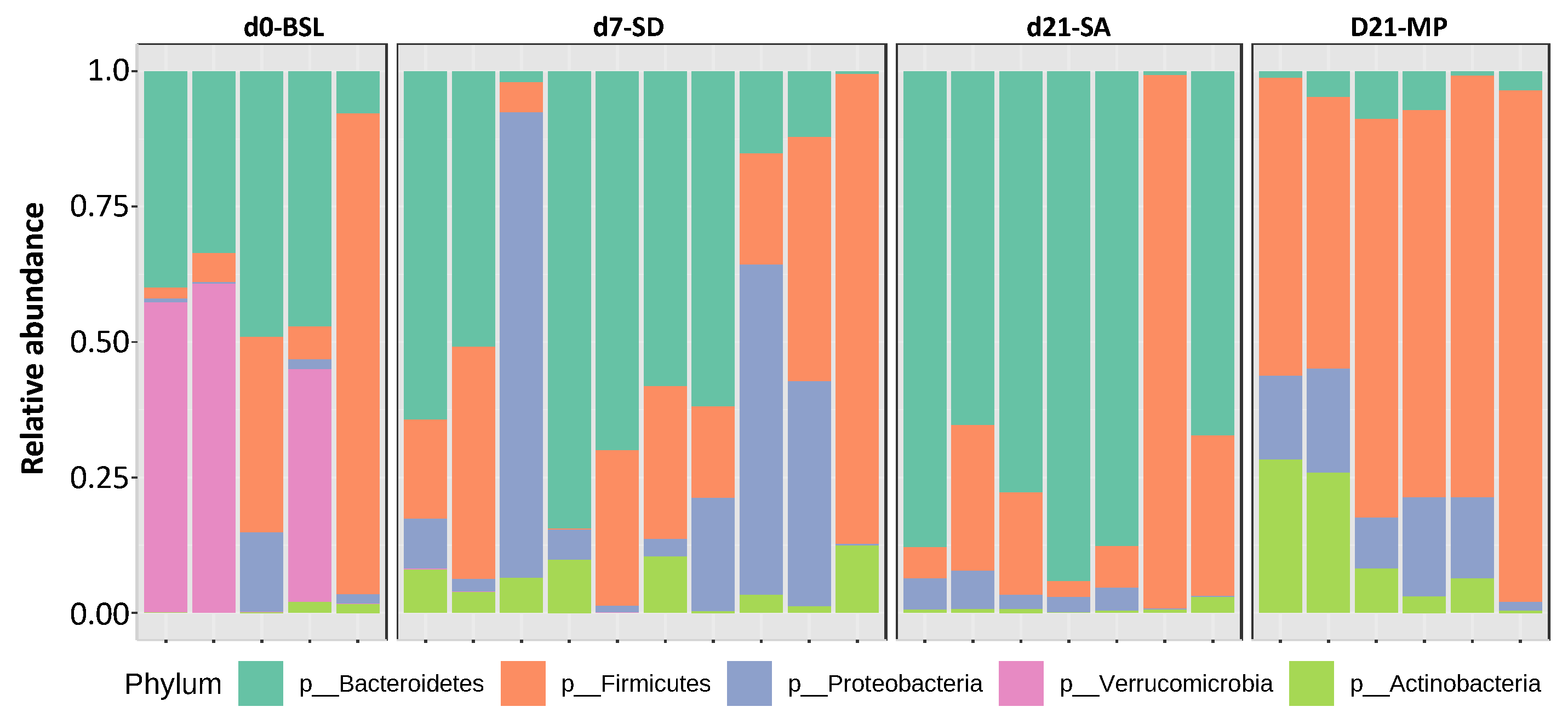

Supplement: Fig. S2 — Changes in the relative abundances of gut bacteria phyla after SD, and 14 days of saline or mixed probiotics gavage. [file spectrum.01437-23-s0002.tiff]
