# Supplementary material for: Sleep deprivation-induced anxiety-like behaviors are associated with alterations in the gut microbiota and metabolites
Source: Microbiol Spectr. 2024 Feb 29;12(4):e01437-23. doi: 10.1128/spectrum.01437-23 (PMC10986621; doi:10.1128/spectrum.01437-23)
Supplement: Supplemental legends — Legends for Fig. S1 and S2 and Tables S1 to S7. [file spectrum.01437-23-s0003.docx]

**Supplemental Figure Legends**

**Supplementary Figure 1. Changes in metabolic functional pathways caused by sleep deprivation.** KEGG pathway enrichment analysis based on significantly (a)

enriched metabolites and (b) reduced metabolites after 7 days of SD.

**Supplementary Figure 2. Changes in the relative abundances of gut bacteria phyla after SD, and 14 days of saline or mixed probiotics gavage.**

**Supplemental Table Legends**

**Supplementary Table 1. The different metabolites between baseline day and 7-day sleep deprivation.**

**Supplementary Table 2. The different metabolites among baseline day, 7-day sleep deprivation and 14-day saline or mixed probiotics supplementation.** BSL: baseline day, d07: after 7-day sleep deprivation, d21-SA: after 14-day saline gavage, d21-MP: after 14-day mixed probiotics gavage.

**Supplementary Table 3. The different metabolites between 7-day sleep deprivation (d7-SD) and 14-day saline supplementation (d21-SA).**

**Supplementary Table 4. The different metabolites between baseline day and 14-day saline supplementation (d21-SA).**

**Supplementary Table 5. The different metabolites between baseline day and 14-day mixed probiotics supplementation (d21-MP).**

**Supplementary Table 6. The spearman’s correlation analysis between movement behaviors and serum metabolites.**

**Supplementary Table 7. The spearman’s correlation analysis between movement behaviors and gut microbes.**
